# S*teinernema sandneri* n. sp. (Rhabditida: Steinernematidae), a new entomopathogenic nematode from Poland

**DOI:** 10.21307/jofnem-2021-051

**Published:** 2021-05-21

**Authors:** Magdalena Lis, Ewa Sajnaga, Marcin Skowronek, Adrian Wiater, Kamila Rachwał, Waldemar Kazimierczak

**Affiliations:** 1 Laboratory of Biocontrol, Production and Application of EPN, Centre for Interdisciplinary Research, Faculty of Natural Sciences and Health, John Paul II Catholic University of Lublin, ul. Konstantynów 1J, 20-708 Lublin, Poland; 2 Department of Industrial and Environmental Microbiology, Faculty of Biology and Biotechnology, Maria Curie-Skłodowska University, ul. Akademicka 19, 20-033 Lublin, Poland; 3 Department of Biotechnology, Microbiology and Human Nutrition, University of Life Sciences in Lublin, ul. Skromna 8, 20-704 Lublin, Poland

**Keywords:** 18S rRNA, D2D3 Domain, Description, Entomopathogenic Nematodes, ITS, Mitochondrial *cox*1, Morphology, Morphometrics, Phylogeny, *Steinernema sandneri*, Taxonomy

## Abstract

A new species of entomopathogenic nematodes, *Steinernema sandneri* n. sp., was recovered by baiting from Poland. Its morphological traits indicate that the new species is a member of the *feltiae-kraussei* group. A body length of 843 (708–965) μm, a more anterior position of excretory pore (56 μm), and the lower D% value (40 vs > 46) discriminate this species from most of the other group members. The first-generation males of *S. sandneri* n. sp. can be distinguished from the other clade members by a 60 μm long spicule, a relatively long gubernaculum (GS% = 79), and the position of the excretory pore (80 μm). Phylogenetic analysis of the ITS rDNA, D2D3 of 28 S rDNA, and *cox*1 sequences confirmed that *S. sandneri* n. sp. is a new species of the *feltiae-kraussei* group, closely related to *S. kraussei* and *S. silvaticum*.

Entomopathogenic nematodes of the families Steinernematidae Travassos, 1927 and Heterorhabditidae Poinar, 1976 are obligate lethal pathogens of insects with a worldwide distribution (Adams et al., 2007; Hominicki, 2002; Spiridonov and Subbotin, 2016). These organisms are commercially produced and used as biological control of insect pest populations (Shapiro-Ilan et al., 2002).

The family Steinernematidae is divided into seven clades: *affine-intermedium*, *bicornutum*, *cameroonense*, *carpocapsae*, *glaseri*, *monticolum*, and *feltiae-kraussei* (Nadler et al., 2006; Spiridonov and Subbotin, 2016). Nematodes of the last group can be characterized by a body length of ≤ 1,000 μm, an elliptical bacterial pouch, and 6-8 lateral fields in infective juveniles (IJs). At present, this group includes *Steinernema kraussei* Steiner, 1923; *S. feltiae* Filipjev, 1934; *S. kushidai* Mamiya, 1988; *S. oregonense* Liu and Berry, 1996; *S. sangi* Phan et al., 2001; *S. weiseri* Mráček et al., 2003; *S. jollieti* Spiridonov et al., 2004a, b; *S. litorale* Yoshida, 2004; *S. akhursti* Qiu et al., 2005; *S. silvaticum* Sturhan et al., 2005; *S. hebeiense* Chen et al., 2006; *S. cholashanense* Nguyen et al., 2008; *S. puntauvense* Uribe-Lorió et al., 2007; *S. texanum* Nguyen et al., 2007; *S. ichnusae* Tarasco et al., 2008; *S. xueshanense* Mráček et al., 2009; *S. citrae* Stokwe et al., 2011; *S. tielingense* Ma et al., 2012a, b; *S. xinbinense* Ma et al., 2012a, b, and *S. nguyeni* Malan et al., 2016. Only 15 of the ~100 recognized species of *Steinernema* have been recorded in Europe so far, including 5 *feltiae-kraussei* representatives: *S. kraussei, S. feltiae*, *S. weiseri* , *S. silvaticum*, and *S. ichnusae*.

In the case of the Steinernematidae, detailed knowledge about the biodiversity and occurrence of this family is important not only scientifically. Since some entomopathogenic nematodes seem to be highly host-specific, every described species of *Steinernema* is a potentially new biological agent assuring more precise and effective control of insect pests. The new *Steinernema* species from Europe is described herein as *S. sandneri* n. sp. on the basis of morphological, morphometric, and molecular data.

*Etymology*: The species is named after Henryk Sandner, zoologist, a pioneer of entomonematology in Poland, Righteous Among the Nations.

## Materials and methods

### Morphological and morphometric studies

For light and scanning electron microscope observations, different life stages of *S. sandneri* were obtained from *infected Galleria mellonella* (Lepidoptera: Pyralidae) larvae exposed individually to ~50 infective juveniles in 0.5 ml Eppendorf test tubes for 18–24 h. Male and female nematodes of the first and second generation were obtained during dissections of insect cadavers in Ringer’s solution after 5 or 10 days at 17.5°C, respectively. IJs were harvested with a modified White trap method (Stock and Goodrich-Blair, 2012) and collected in tap water for 5 days after initial migration. For light microscopy, all developmental stages of the nematodes were heat-relaxed in Ringer’s solution (55°C, 5 min) and fixed in 2% formalin (48 h, room temperature). After fixation, the specimens were processed using the modified Seinhorst (1959) method and mounted in pure glycerin. All measurements were performed with a Leica 5500B microscope fitted with DIC optics, a digital camera (Leica 290HD), and the Leica Application Suite ver. 3.8.0 software. For SEM of IJs, first-generation males and females of the nematodes were prepared as described previously by Skrzypek et al., 2011 and observed with a scanning electron microscope (LEO 1430VP) at 15-kV accelerating voltage in a high-vacuum mode.

### Hybridization test

Reproductive isolation of *S. sandneri* (isolate S17-050) and *S. kraussei*, *S. silvaticum*, *S. feltiae*, *S. oregonense*, *S. ichnusae*, *S. weiseri*, *S. jollieti*, and *S. cholashanense* was tested using the Nguyen and Duncan (2002) method. Simultaneously, negative (virginity/self-fertilization) and positive (crosses between females and males of the same species) controls were performed. All the treatments were replicated 30 times for each combination of the nematode species and observed for 20 consecutive days at 17.5°C.

### Molecular characterization and phylogenetic analysis

DNA was extracted from three single virgin first-generation females of nematodes using a DNeasy Blood and Tissue Kit (Qiagen, Germany). PCR amplification of the internal transcribed spacer (ITS) region of rDNA, the D2D3 region of 28 S rDNA, and the mitochondrial *cox*1 gene encoding cytochrome c oxidase subunit was performed as described earlier by Lis et al. (2019). Three sets of primers (synthesized by Genomed, Poland) were used: 18 S and 26 S for ITS (Vrain et al., 1992), D2F and 536 F for D2D3 (Nguyen, 2007b; Stock et al., 2001), and 507 F and 588 R for *cox*1 gene (Nadler et al., 2006). The sequences obtained in this study were compared with those deposited in the GenBank using BLAST available on the NCBI website. Multiple sequence alignments were created using ClustalW (Higgins and Sharp, 1988) at the default configuration included in MEGA 6.06 (Tamura et al., 2013) and then optimized manually. Based on the aligned sequence datasets, phylogenetic trees of the studied nematode strains were inferred in MEGA 6.06 using the Maximum Likelihood method with best fit nucleotide base substitution models HKY + G for ITS, GTR + G for D2D3, and HKY + G + I for the *cox*1 gene (Hasegawa et al., 1985; Nei and Kumar, 2000). *Caenorhabditis elegans* was used as an outgroup. To determine the statistical support for the branches, bootstrapping with 1,000 replicates of the data were conducted (Felsenstein, 1985). Percentages of sequence identity were calculated from the multiple alignments using the SIAS (Sequence Identity and Similarity) application at the default configuration (Reche, 2008). Estimation of evolutionary divergence expressed by the number of base differences between the sequences was performed using Mega 6.06 at the pairwise deletion option. The number of unique positions in the sequences of *S. sandneri* S17-050 was computed using the same program. Accessions numbers of all sequences and details on nematode taxa used in the molecular study are presented in Table S1.

**Table S1.** Details on taxa used in the molecular analyses.

		GeneBank accession no.		
Species	Isolate name/geographic origin	ITS rDNA	28S rDNA	*cox*1
***Steinernema sandneri*** **n. sp**.	**S17-050, Poland**	**MW078536**	**MW078535**	**MW078544**
*Steinernema affine*	B1, England		AF331899	
*Steinernema affine*	The Netherlands	AY171298		
*Steinernema akhursti*	China	DQ375757		
*Steinernema bicornutum*	Serbia		AF331904	
*Steinernema bicornutum*	Yugoslavia	AF121048		
*Steinernema cameroonense*	OB, Cameroon	JX985267		
*Steinernema carpocapsae*	Russia	AY171282		
*Steinernema cholashanense*	Tibet, China	EF431959	EF520284	
*Steinernema citrae*	141-C, South Africa	EU740970	GU004534	
*Steinernema costaricense*	Costa Rica		EF187017	
*Steinernema feltiae*	Bodega Bay, USA		AF331906	
*Steinernema feltiae*	SN, USA	AF121050		
*Steinernema feltiae*	3, Portugal			JQ423217
*Steinernema glaseri*	NC, USA		AF331908	
*Steinernema glaseri*	NJ, USA	AF122015		
*Steinernema hebeiense*	G6, China	DQ105794		
*Steinernema hermaphroditum*	VK-2013, India	KC252604		
*Steinernema ichnusae*	Sardinia, Italy	EU421129	EU421130	
*Steinernema jollieti*	Monsanto, USA		GU569051	GU569068
*Steinernema jollieti*	73, USA	AY171265		
*Steinernema kraussei*	Westphalia, Germany	AY230175	AF331896	AY943990
*Steinernema kraussei*	Altai 35, Russia	AY171270		
*Steinernema kraussei*	Nash, UK	AY230176		
*Steinernema kraussei*	Italy	AY230174		
*Steinernema kraussei*	Iceland	AY171248		
*Steinernema kraussei*	20F, Portugal	JN683825		
*Steinernema kraussei*	D, Switzerland	AY171258		
*Steinernema kraussei*	Russia	AY171264		
*Steinernema kraussei*	HkHm22, Japan	AB243442		
*Steinernema kraussei*	Skr-LUB, Lublin, Poland	KY819012		
*Steinernema kraussei*	B2, UK	AY230161		
*Steinernema kraussei*	20F, Portugal			JN683829
*Steinernema kraussei*	Quebec, Canada		GU569053	
*Steinernema kraussei*	SKR S11-50, Poland	MW647848	MW647849	MW647850
*Steinernema kushidai*	Hamakita, Japan	AB243440		
*Steinernema kushidai*	N22, Japan			AY943991
*Steinernema kushidai*	Japan		AF331897	
*Steinernema litorale*	AiAt199, Japan	AB243441		
Species	Isolate name/geographic origin	GeneBank accession no.		
		ITS rDNA	28S rDNA	*cox*1
*Steinernema monticolum*	Korea, South Korea	AF122017	EF439651	
*Steinernema monticulum*	Mt. Chiri, South Korea			AY943994
*Steinernema nguyeni*	F2, South Africa	KP325084		
*Steinernema oregonense*	Oregon, USA	AF122019		
*Steinernema oregonense*	OS-10, USA		AF331891	AY943995
*Steinernema sangi*	Vietnam	AY355441	GU569057	
*Steinernema scarabaei*	New Jersey, USA		AY172023	
*Steinernema scarabaei*	Chile	FJ263673		
*Steinernema silvaticum*	S16/019, Poland	MG543845	MG547576	MG547572
*Steinernema silvaticum*	B, Germany	AY171255		
*Steinernema silvaticum*	B3, UK (type)	AY230162		
*Steinernema texanum*	Texas, USA	EF152568	EF152569	
*Steinernema tielingense*	LFS65, China	GU994201	GU994202	
*Steinernema weiseri*	F, Germany	AY171268		
*Steinernema weiseri*	Turkey		GU569059	GU569075
*Steinernema xinbinense*	LFS8, China	JN171593		
*Steinernema xinbinense*	LFS40, China		GU994202	
*Steinernema xueshanense*	Yunnan, China	FJ666052	FJ666053	
*Caenorhabditis elegans*	N2 Bristol, USA			NC001328
*Caenorhabditis elegans*		X03680	X03680	

## Results

### Systematics

*Steinernema sandneri* n. sp.

LSID: 051B950B-081C-4FD9-A8C2-22E7106C29BE.

(Figures 1–8; Tables 1–6).

### Description

#### Infective juvenile

**Figure 1:**
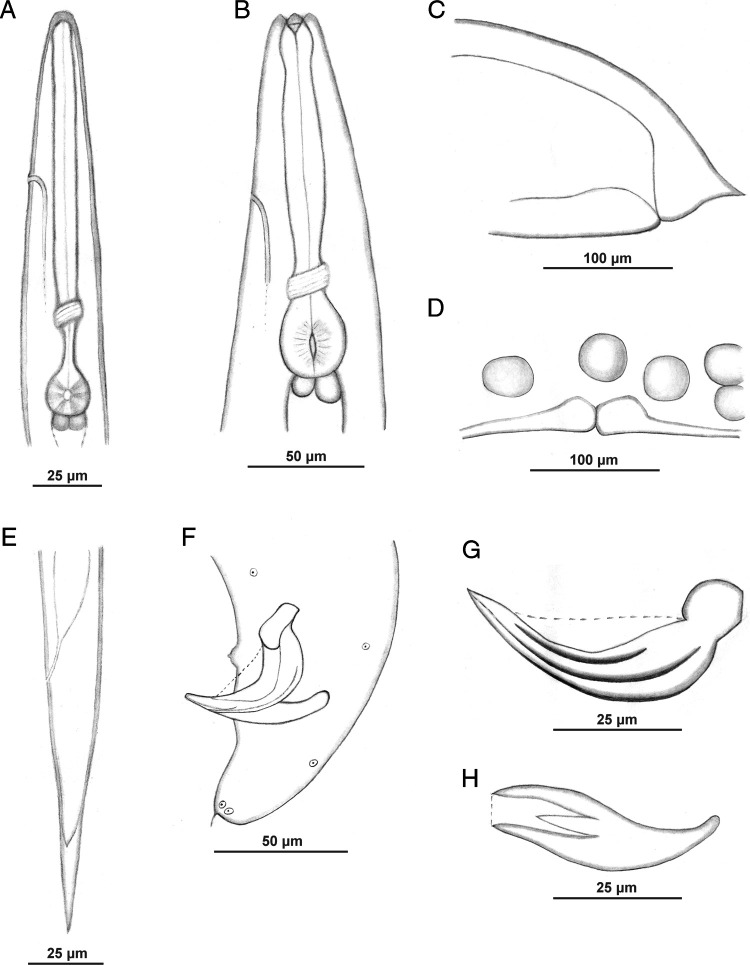
*Steinernema sandneri* n. sp. A: infective juvenile, anterior region; B: first-generation male, anterior region; C: first-generation female, tail region; D: first-generation female, vulval region; E: infective juvenile, tail region; F: first-generation male, tail region; G: spicule; H: gubernaculum. Scale bars as on images. Lateral views.

**Figure 2:**
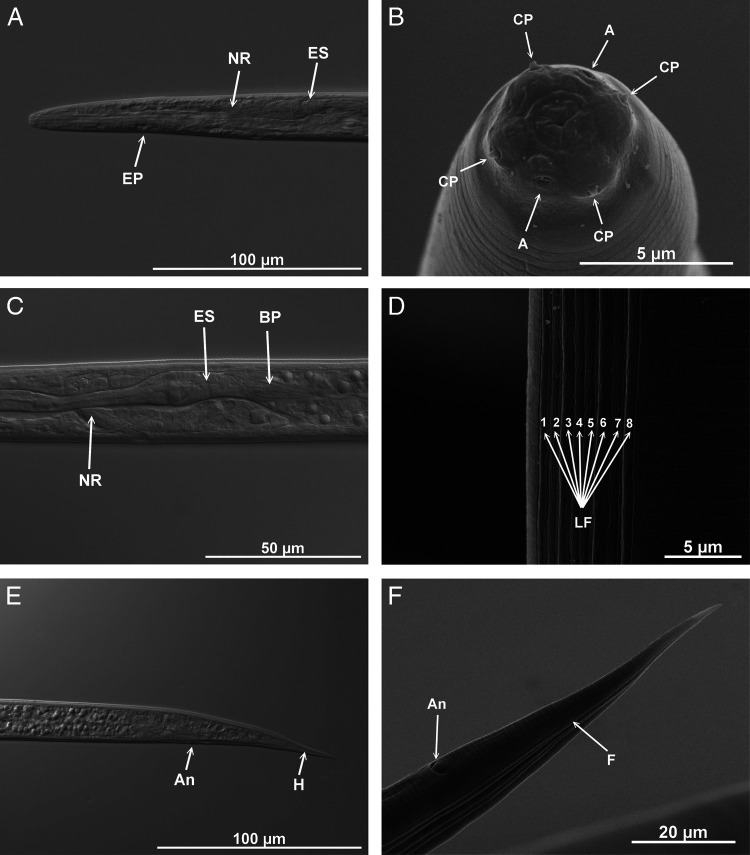
*Steinernema sandneri* n. sp. Differential interference contrast (A,C,E) and scanning electron (B,D,F) micrographs of infective juveniles. A – amphid openings, An – anus, BP – bacterial pouch, CP – cephalic papillae, EP – excretory pore, ES – esophagus, F – phasmid opening, H – hyaline part, LF – lateral fields, NR – nerve ring. Scale bars as on images.

Body straight or slightly abdominally curved when heat-relaxed, tapering gradually from the base of esophagus to the anterior end and from anus to the distal end. Second-stage cuticle present shortly after leaving the host body, with six labial and four cephalic papillae, but lost in storage after a few days/weeks (depending on the temperature). Cephalic region continuous with body smooth, truncate-conical, with four cephalic papillae and prominent amphidal apertures (Figs 1A and 2B). Mouth and anus closed (Fig. 2B,F). Cuticle with prominent striation along almost the whole body (Fig. 2B,D,F). Lateral fields beginning as a single line close to the anterior end, increasing to eight ridges, posteriorly gradually reduced to four (anus level) and two (phasmid level) (Fig. 2D,F). Deirids not visible. Esophagus with narrow corpus, slightly swollen metacorpus, isthmus surrounded by nerve ring (Fig. 2A,C). Excretory pore in the middle between anterior end and basal bulb (Figs 1A and 2A). Hemizonid distinct, between nerve ring and esophagus base. Cardia present. Bacterial vesicle well developed, with visible rod-shaped bacteria (Fig. 2C). Tail conical, tapering gradually. Phasmids distinct, located 40% of tail length, posterior to anus. Hyaline portions comprising ca. 1/3 of tail length (Figs 1E and 2E,F).

#### First-generation male

**Figure 3.**
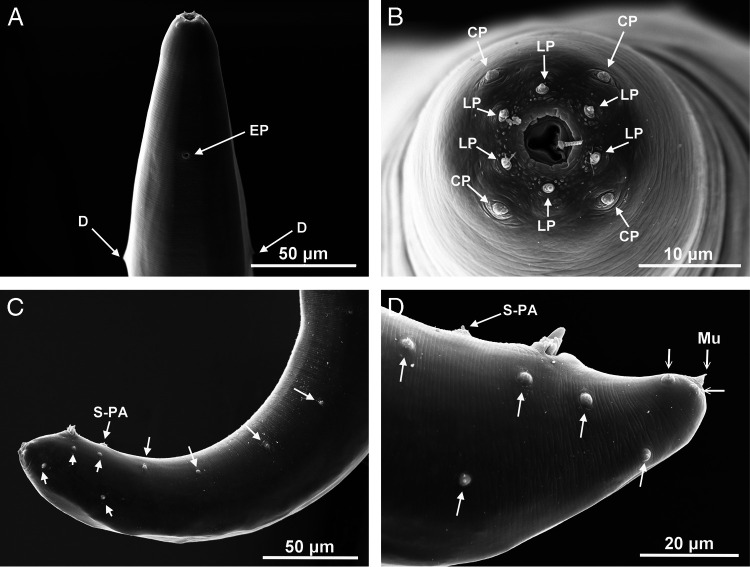
*Steinernema sandneri* n. sp. Scanning electron micrographs of first-generation males. A,B: anterior region with excretory pore (EP), deirids (D), cephalic (CP) and labial papillae (LP). C,D: posterior region with genital papillae (arrows), single preanal papilla (S-PA) and mucron (Mu). Scale bars as on images.

**Figure 4:**
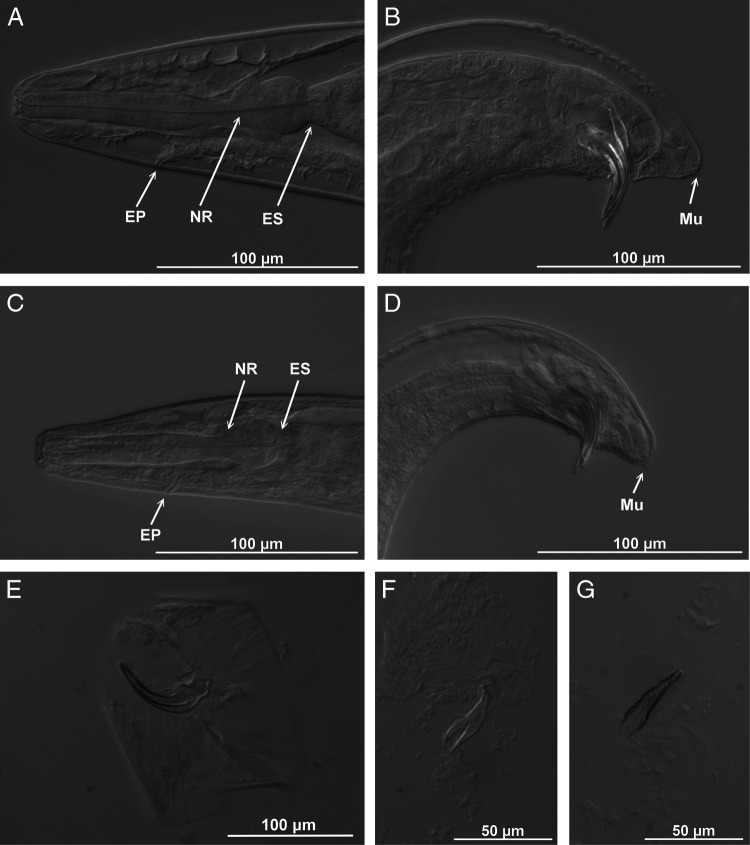
*Steinernema sandneri* n. sp. Differential interference contrast micrographs of first- (A,B,E,F,G) and second-generation males (C,D). A,C: anterior region with esophagus (ES), excretory pore (EP) and nerve ring (NR). B,D: posterior region with spicules, gubernaculum, and mucron (Mu). E: spicules. F,G: gubernaculum (lateral and ventral view). Scale bars as on images.

Body C- or J-shaped when heat-relaxed. Cuticle with faint transverse striation visible in SEM (Fig. 3A–D). Lateral fields not observed. Cephalic region smooth, rounded, with four cephalic and six smaller labial papillae and slit-like amphid openings (Fig. 3A,B). Stoma shallow, funnel-shaped, cheilorhabdions prominent. Esophagus with cylindrical procorpus, slightly swollen metacorpus, and narrower isthmus surrounded by nerve ring located anteriorly to basal bulb. Excretory pore anterior to nerve ring, close to metacorpus (Figs 1B and 4A). Cardia prominent. Anterior deirids similar to genital papillae in shape and size (Fig. 3A). Posterior deirids usually located anteriorly, just before the first pair of genital papillae. Testis monorchic, reflexed. Spicule with two ribs and velum not reaching spicule tip (Figs 1F,G, 4E). Gubernaculum boat-shaped in lateral view, with ventrally curved manubrium (Figs 1H and 4F,G). Typically 23 genital papillae present, comprising 11 pairs and 1 single precloacal midventral. Additional papillae – if occur – usually before posterior deirids. Phasmid openings between ventral last pair of genital papillae. Tail terminus with mucron (Figs 1F, 3D and 4B).

#### Second-generation male

Similar to first-generation male but shorter and more slender. Excretory pore located more posteriorly. Tail relatively longer, with prominent mucron (Table 1; Fig. 4C,D).

**Table 1.** Morphometrics (in μm) of different developmental stages of *Steinernema sandneri* n. sp. [mean ± SE (range)] [*N* = 25].

	First generation			Second generation		
	Males		Females	Males	Females	Infective juveniles
Character	Holotype	Paratypes	Paratypes	Paratypes	Paratypes	Paratypes
Body length [L]	1,565.3	1,461 ± 22.1 (1,205.7–1,635.3)	4,628 ± 46.4 (4,244.0–5,014.0)	946 ± 13.8 (817.5–1,093.8)	2,120 ± 51.5 (1,640.6–2,753.2)	843.0 ± 13.9 (708.2–964.5)
Greatest body width [W]	143.0	155.1 ± 2.7 (123.8–177.7)	209.6 ± 3.4 (181.3–261.3)	70.1 ± 1.1 (54.9–79.5)	126.6 ± 3.3 (88.9–146.6)	27.4 ± 0.5 (23.0–31.9)
Anterior end to excretory pore [EP]	88.4	80.4 ± 1.5 (63.5–92.4)	84.4 ± 2.1 (61.4–101.6)	69.8 ± 1.5 (59.0–84.6)	72.1 ± 1.3 (57.3–88.4)	55.9 ± 0.8 (44.4–64.2)
Anterior end to nerve ring [NR]	121.9	126.0 ± 1.5 (112.0–138.1)	146.7 ± 1.2 (132.5–157.6)	97.7 ± 0.8 (86.4–105.8)	113.7 ± 1.1 (102.5–124.6)	102.6 ± 1.4 (82.6–117.9)
Anterior end to esophagus [ES]	155.9	157.2 ± 1.1 (147.6–169.6)	184.7 ± 1.0 (173.2–193.9)	120.5 ± 1.1 (109.0–128.7)	145.8 ± 1.4 (130.3–158.5)	138.4 ± 0.5 (122.5–150.5)
Testis reflection	461.6	452.1 ± 9.5 (359.3–537.7)	–	202.6 ± 13.2 (84.9–379.2)	–	–
Tail length [T]	45.1	41.2 ± 0.5 (35.4–45.5)	46.7 ± 1.6 (32.4–60.9)	42.3 ± 1.0 (31.7–52.1)	57.5 ± 1.4 (46.5–72.1)	75.2 ± 1.1 (64.4–86.4)
Anal body diameter [ABW]	50.1	54.1 ± 0.6 (49.9–59.2)	94.0 ± 2.9 (62.1–121.8)	36.8 ± 0.4 (30.7–40.8)	54.4 ± 1.5 (43.1–70.7)	17.3 ± 0.4 (14.6–23.8)
Spicule length [SL]	64.2	59.8±0.5 (52.6–65.3)	–	51.2 ± 0.9 (42.5–60.2)	–	–
Gubernaculum length [GL]	39.2	43.6 ± 0.5 (39.1–50.2)	–	30.4 ± 0.6 (24.2–39.5)	–	–
a [L/W]	10.9	9.5 ± 0.1 (8.5–11.0)	22.2 ± 0.4 (17.4–24.7)	13.6 ± 0.2 (12.0–16.4)	16.9 ± 0.4 (14.1–23.2)	30.9 ± 0.3 (27.2–33.8)
b [L/ES]	10.4	9.3 ± 0.1 (8.0–10.2)	25.1 ± 0.2 (23.5–27.2)	7.9 ± 0.1 (7.2–9.4)	14.5±0.3 (12.0–18.1)	6.1 ± 0.1 (5.5–6.9)
c [L/T]	34.7	35.6 ± 0.5 (31.2–41.9)	102.0 ± 3.8 (75.4–140.3)	22.7 ± 0.6 (17.2–28.5)	37.3 ± 1.1 (24.6–50.1)	11.2 ± 0.1 (10.5–13.2)
Hyaline% [(H/T) × 100]	–	–	–	–	–	33.6 ± 3.9 (22.7–39.9)
D% [(EP/ES) × 100]	56.7	51.2 ± 0.9 (42.1–59.3)	45.7 ± 1.1 (35.5–54.2)	58.0±1.2 (48.1–71.9)	49.5 ± 0.9 (36.2–58.1)	40.4 ± 0.4 (35.8–44.8)
E% [(EP/T) × 100]	196.0	195.8 ± 4.0 (160.2–240.9)	186.0 ± 7.9 (128.1–266.8)	167.4 ± 1.2 (128.2–222.8)	127.2 ± 3.9 (101.4–163.9)	74.4 ± 0.9 (62.6–85.8)
SW% [(SL/ABW) × 100]	128.1	110.9 ± 1.5 (97.0–126.9)	–	139.6 ± 3.2 (105.1–171.4)	–	–
GS% [(GL/SL) × 100]	61.1	79.1 ± 1.2 (60.8–82.8)	–	59.6 ± 1.1 (49.5–68.6)	–	–
V% [(Vulva – anterior end/L) × 100]	–	–	53.7 ± 0.3 (49.0–56.8)	–	54.3 ± 0.8 (39.3–59.1)	–

Note: – = character absent.

#### First-generation female

**Figure 5:**
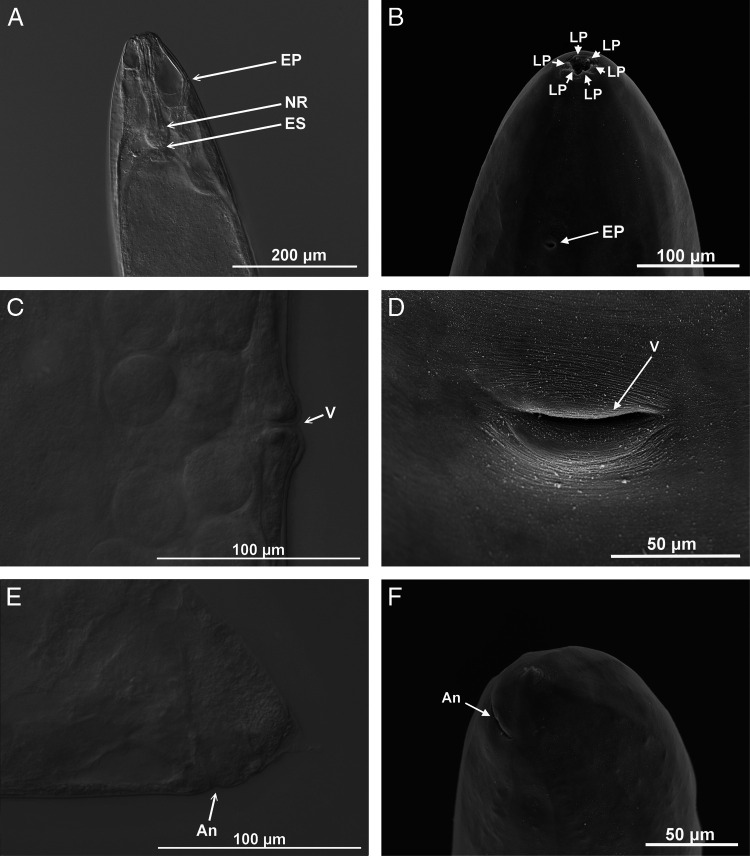
*Steinernema sandneri* n. sp. Differential interference contrast (A,C,E) and scanning electron (B,D,F) micrographs of first-generation females. An – anus, EP – excretory pore, ES – esophagus, LP – labial papillae, NR – nerve ring, V – vulva. Scale bars as on images.

Body C-shaped when heat-relaxed and fixed. Cuticle smooth when observed in a light microscope, with faint striation in SEM (Fig. 5B,D,F). Lateral fields not observed. Deirids inconspicuous, difficult to observe even under SEM. Labial region rounded, continuous with the body. Six labial and four cephalic papillae (Fig. 5B). Slit-like amphidial apertures. Cheliorhabdions large, sclerotized. Stoma prominent. Esophagus with cylindrical procorpus, swollen metacorpus, and distinct isthmus. Excretory pore in mid-esophagus region. Nerve ring just anterior to basal bulb (Fig. 5A). Cardia prominent. Gonads amphidelphic, reflexed. Vulva in the form of transverse slit located slightly posterior to mid-body (Table 1). Vulval lips slightly protruding, asymmetrical, with larger posterior lip (Figs 1D and 5C,D). Tail length shorter than body anal diameter, with slight post-anal swelling. Tail terminus with mucron (Figs 1C and 5E,F).

#### Second-generation female

Similar to first-generation female but smaller. Vulva more protruding, with distinct asymmetry between lips. Tail with mucron, but without pronounced post-anal swelling (Table 1).

#### Life cycle

*Steinernema sandneri* n. sp. can be successfully reared on *G. mellonella* or *Tenebrio molitor* (Coleoptera: Tenebrionidae) larvae at a temperature in the range of 15–20°C. The life cycle of *S. sandneri* n. sp. is similar to that of other *Steinernema* species. *G. mellonella* larvae exposed to 50–100 IJs die within 3–4 days. Adults of the second generation can be found in insect cadaver 8–12 days after infection. Pre-infective juveniles migrate from the host body, mature for a few days, and migrate to water trap after 18–21 days.

#### Cross-breeding tests

Mating attempts were observed between *S. sandneri* n. sp. and *S. kraussei* and *S. silvaticum*, but no fertile offspring was produced in any of the crosses. Hybridization tests with *S. kraussei*, *S. silvaticum*, *S. feltiae*, *S. oregonense*, *S. ichnusae*, *S. weiseri*, *S. jollieti*, and *S. cholashanense* showed that *S. sandneri* n. sp. was reproductively isolated. The positive control always yielded a progeny.

#### Diagnosis and relationship

The new species was characterized by analysis of the morphology and morphometrics of IJs and adults (Table 1). IJs have a body length of 843 μm (708–965), a body diameter of 27 (23–32) μm, and a tail length of 75 (64–86) μm. The distance from the anterior end to the excretory pore is 56 (44–64) μm and to the esophagus base is 138 (123–151) μm, D% = 40 (36–45), E% = 74 (63–86). The lateral field in the mid-body region has 8 ridges, and the hyaline part of the tail occupies ~1/3 of its length. The first-generation male is characterized by a spicule length of 60 (53–65) μm and by a gubernaculum length of 44 (39–50) μm. The spicule manubrium is almost as long as it is wide, the shaft is short, and the velum expands from the calomus to the end of the ventral rib. The tail of both generation males is mucronated. The first- and second-generation females of *S. sandneri* n. sp. have a slightly protruding vulva and a mucron at the posterior end.

The species belongs to the *feltiae-kraussei* group of *Steinernema*, which comprises over 20 species. The length of *S. sandneri* n. sp. IJs 843 μm (708–965) is typical for this group, identical with the length *S. cholashanense* 843 μm (727–909), and similar to that of *S. feltiae* 849 μm (766–928), *S. silvaticum* 860 μm (670–975), *S. xueshanense* 860 μm (768–929), and *S. ichnusae* 866 μm (767–969). The IJs of *S. sandneri* n. sp. can be distinguished from these species by the relatively thicker body a = 6.1 (5.5–6.9) vs > 6.3 (5.6–7.7), the more anteriorly located excretory pore 56 μm (44–64) vs >62 μm (51–73), and the lower D% value 40 (36–45) vs > 46 (42–53). The position of the IJ nerve ring 103 μm (83–118) is more anterior than in *S. feltiae* 113 μm (108–117). The esophagus of *S. sandneri* n. sp. IJs is longer 138 μm (123–151) than in most of the clade species except for *S. ichnusae* 138 μm (119–148) (Table 2).

**Table 2.** Comparative morphometrics of third-stage infective juveniles of *S. sandneri* n. sp. and related *Steinernema* spp.

	Morphometric character^a^											
Species	L	W	EP	NR	ES	T	a	b	c	D%	E%	Reference
*S. kushidai*	589 (424–662)	26 (22–31)	46 (42–50)	76 (70–84)	111 (106–120)	50 (44–59)	22.5 (19–25)	5.3 (4.9–5.9)	11.7 (10–13)	41 (38–44)	92 (NA)	Mamiya, (1988)
*S. hebeiense*	658 (610–710)	26 (23–28)	48 (43–51)	78 (73–83)	107 (100–111)	66 (63–71)	26 (24–28)	6.2 (5.7–6.7)	10 (9.4–11)	45 (40–50)	72 (65–80)	Chen et al. (2006)
*S. puntauvense*	670 (631–728)	33 (31–38)	25 (20–30)	54 (46–69)	94 (81–103)	54 (51–59)	20 (17–23)	6.1 (7.1–7.9)	12 (11–13)	42 (25–50)	44 (35–56)	Uribe–Lorío et al. (2007)
*S. xinbinense*	694 (635–744)	30 (28–31)	51 (46–53)	86 (75–90)	116 (109–125)	73 (65–78)	24 (21–25)	6.1 (5–7)	9.7 (8–11)	44 (40–47)	71 (65–78)	Ma et al. (2012)
*S. jollieti*	711 (625–820)	23 (20–28)	60 (53–65)	NA	123 (115–135)	68 (60–73)	31 (25–34)	5.7 (4.9–6.4)	10.5 (9.0–11.7)	48 (46–50)	88 (NA)	Spiridonov et al. (2004b)
*S. nguyeni*	737 (673–796)	25 (22–28)	52 (47–58)	80 (74–86)	110 (101–121)	67 (61–73)	29 (27–33)	6.7 (6.2–7.4)	11 (10–12)	48 (43–57)	79 (70–86)	Malan et al. (2016)
*S. weiseri*	740 (586–828)	25 (24–29)	57 (43–65)	84 (72–92)	113 (95–119)	60 (49–68)	29 (25–33)	6.6 (5.7–7.2)	12 (10–14)	51 (44–55)	95 (NA)	Mráček et al. (2003)
*S. sangi*	753 (704–784)	35 (30–40)	52 (46–54)	91 (78–97)	127 (120–138)	81 (76–89)	22 (19–25)	5.9 (5.6–6.3)	9.3 (8.7–10.2)	40 (36–44)	62 (56–70)	Phan et al. (2001)
*S. citrae*	754 (623–849)	26 (23–28)	56 (49–64)	98 (83–108)	125 (118–137)	71 (63–81)	30 (25–34)	6.0 (5.1–7.1)	15 (13–14)	44 (39–58)	110 (85–132)	Stokwe et al. (2011)
*S. texanum*	756 (732–796)	30 (29–34)	59 (52–62)	92 (84–102)	115 (111–120)	73 (60–79)	25 (22–27)	6.5 (6.2–7.0)	10 (9.6–12.5)	51 (46–53)	81 (76–88)	Nguyen et al. (2007)
*S. akhursti*	812 (770–835)	33 (33–35)	59 (55–60)	90 (83–95)	119 (115–123)	73 (68–75)	24 (23–26)	6.8 (6.6–7.2)	11 (10–12)	47 (45–50)	77 (73–86)	Qiu et al. (2005)
***S. sandneri*** **n. sp.**	**843 (708**–**965)**	**27 (23**–**32)**	**56 (44**–**64)**	**103 (83**–**118)**	**138 (123**–**151)**	**75 (64**–**86)**	**31 (27**–**34)**	**6.1 (5.5**–**6.9)**	**11.2 (11**–**13.2)**	**40 (36**–**45)**	**74 (63**–**86)**	–
*S. cholashanense*	843 (727–909)	30 (26–35)	62 (59–65)	87 (72–97)	125 (110–138)	73 (60–80)	28 (24–34)	6.8 (6.1–7.2)	12 (10–14)	49 (46–53)	81 (76–91)	Nguyen et al., (2008)
*S. feltiae*	849 (766–928)	29 (22–32)	63 (58–67)	113 (108–117)	136 (130–143)	86 (81–89)	30 (27–34)	6.4 (5.8–6.8)	10 (9.4–11)	46 (44–50)	74 (67–81)	Nguyen et al., (2007)
*S. silvaticum*	860 (670–975)	30 (26–35)	62 (51–73)	96 (75–109)	121 (100–141)	75 (63–86)	29 (23–33)	7.3 (6.3–7.7)	11.4 (9.9–13.1)	50 (46–56)	–	Sturhan et al. (2005)
*S. xueshanense*	860 (768–929)	30 (29–33)	67 (60–72)	91 (81–96)	135 (130–143)	87 (80–92)	28 (26–32)	6.4 (5.8–7.0)	9.9 (9.0–11)	50 (46–52)	78 (70–90)	Mrácˇek et al. (2009)
*S. ichnusae*	866 (767–969)	31 (27–35)	63 (59–68)	102 (94–108)	138 (119–148)	81 (76–89)	28 (24–32)	6.3 (5.6–6.9)	11 (8.8–12)	46 (42–49)	77 (68–83)	Tarasco et al. (2008)
*S. litorale*	909 (834–988)	31 (28–33)	61 (54–69)	96 (89–104)	125 (114–133)	83 (72–91)	29.5 (27–31)	7.3 (6.7–7.9)	11 (9.7–11.9)	49 (44–56)	73 (68–84)	Yoshida (2004)
*S. tielingense*	915 (824–979)	35 (32–38)	69 (64–73)	98 (90–105)	128 (120–135)	81 (74–85)	26 (23–28)	7 (6–8)	11 (9–13)	55 (47–61)	88 (85–94)	Ma et al. (2012)
*S. kraussei*	951 (797–1,102)	33 (30–36)	63 (50–66)	105 (99–111)	134 (119–145)	79 (63–86)	29 (NA)	7.1 (NA)	12.1 (NA)	47 (NA)	80 (NA)	Nguyen et al. (2007)
*S. oregonense*	980 (820–1,110)	34 (28–38)	66 (60–72)	NA	132 (116–148)	70 (64–78)	30 (24–37)	7.6 (6–8)	14 (12–16)	50 (40–60)	100 (90–110)	Liu and Berry (1996)

Notes: Measurements are given in μm and in the form: mean (range). ^a^abbreviations as in Table 1, NA = data not available.

First-generation males of S*. sandneri* n. sp. can be distinguished from other *feltiae-kraussei* clade members by shorter spicules 60 μm (53–65), except of *S. hebeiense* 57 μm (51–63), *S. xinbinense* 56 μm (49–62), *S. silvaticum* 51 μm (42–64), and *S. kraussei* 49 μm (42–53). The SW% value of *S. sandneri* n. sp. males 111 (97–127) is similar only to that of *S. feltiae* 113 (99–130), *S. kraussei* 110 (range: data not available), and *S. cholashanense* 115 (92–144). The relative length of the gubernaculum is high GS% = 79 (61–83) and comparable only with *S. oregonense* GS% = 79 and *S. weiseri* GS% = 80 (70–85). The position of the excretory pore in *S. sandneri* n. sp. males D% = 51 (42–59) is more anterior than in most clade species, with the exception of *S. hebeiense* D% = 51 (48–59), *S. nguyeni* D% = 48 (38–57), *S. weiseri* D% = 49 (39–60), *S. sangi* D% = 49 (42–63), and *S. kraussei* D% = 53 (range: data not available). The mucron in first-generation males distinguishes S. *sandneri* n. sp. from European *S. weiseri*, *S. ichnusae*, and *S. silvaticum* (Table 3).

**Table 3.** Comparative morphometrics of first-generation males of *S. sandneri* n. sp. and related *Steinernema* spp.

	Morphometric character^a^							
Species	SL	GL	W	D%	SW%	GS%	MUC^b^	n
***S. sandneri*** **n. sp.**	**60 (53**–**65)**	**44 (39**–**50)**	**155 (124**–**178)**	**51 (42**–**59)**	**111 (97**–**127)**	**79 (61-**–**83)**	**P**	**25**
*S. akhursti*	90 (85–100)	64 (58–68)	131 (115–150)	56 (52–61)	180 (140–200)	71 (65–77)	P	20
*S. cholashanense*	66 (60–71)	39 (32–45)	137 (73–204)	64 (50–85)	115 (92–144)	71 (61–85)	P	20
*S. citrae*	65 (57–80)	44 (32–59)	103 (87–113)	58 (47–67)	198 (156–233)	68 (48–89)	P	20
*S. costaricense*	92 (81–101)	46 (41–51)	128 (89–157)	53 (51–66)	160 (150–170)	49 (45–55)	A	19
*S. feltiae*	70 (65–77)	41 (34–47)	75 (60–90)	60 (51–64)	113 (99–130)	59 (52–61)	P	25
*S. hebeiense*	57 (51–63)	46 (38–50)	86 (74–98)	51 (48–59)	140 (120–170)	80 (60–90)	A	20
*S. ichnusae*	66 (64–67)	44 (43–46)	137 (73–204)	62 (59–65)	139 (120–162)	67 (64–69)	A	20
*S. jollieti*	64 (55–70)	54 (45–60)	115 (98–135)	64 (53–83)	145 (NA)	84 (NA)	A	12
*S. kraussei*	49 (42–53)	33 (29–37)	128 (110–144)	53 (NA)	110 (NA)	67 (NA)	P	NA
*S. kushidai*	63 (48–72)	44 (39–60)	97 (75–156)	51 (42–59)	150 (NA)	70 (NA)	A	20
*S. litorale*	75 (67–89)	53 (44–64)	96 (82–111)	40 (34–56)	174 (154–200)	71 (62–81)	P	25
*S. nguyeni*	66 (58–75)	43 (30–55)	82 (58–106)	48 (38–57)	215 (185–279)	66 (46–81)	P	20
*S. oregonense*	71 (65–73)	56 (52–59)	138 (105–161)	73 (64–75)	151 (NA)	79 (NA)	A	20
*S. puntauvense*	77 (71–81)	34 (30–40)	119 (101–139)	67 (45–85)	170 (140–200)	65 (55–75)	P	19
*S. sangi*	63 (58–80)	40 (34–46)	159 (120–225)	49 (42–63)	150 (120–160)	60 (50–70)	P	20
*S. silvaticum*	51 (42–64)	37 (30–43)	65 (52–78)	60 (45–63)	NA	NA	P	26
*S. texanum*	60 (55–66)	45 (39–53)	99 (81–116)	67 (58–73)	157 (127–203)	75 (62–84)	A	20
*S. tielingense*	88 (79–98)	62 (49–70)	129 (111–159)	71 (64–78)	191 (176–212)	73 (59–82)	A	20
*S. weiseri*	68 (62–72)	53 (46–57)	112 (84–138)	49 (39–60)	180 (150–240)	80 (70–85)	A	20
*S. xinbinense*	56 (49–62)	35 (30–41)	103 (90–126)	45 (41–50)	137 (114–156)	63 (54–72)	P	20
*S. xueshanense*	76 (66–91)	49 (41–60)	144 (97–159)	80 (73–87)	152 (93–172)	64 (58–95)	A	20

Notes: Measurements are given in μm and in the form: mean (range). ^a^abbreviations as in Table 1. ^b^MUC = mucron; P = present, A = absent, NA = data not available.

#### Type locality and habitat

Natural host unknown. The nematode isolate S17-050 was obtained from sandy-loamy soil samples collected in eastern Poland (51°46’55”N 22°42’35”, 147 m a.s.l.) in 2017. The soil samples were collected in a mixed forest from 0 to 20 cm depth. Nematodes were isolated using a modified live trap method (Bedding and Akhurst, 1975) with the use of *G. mellonella* larvae as a bait. Detailed studies were performed on a straight line of nematodes (offspring of 2 IJs) reproducing successfully in *G. mellonella* and maintained in our laboratory.

#### Type designation and deposition

Holotype male, paratype males, paratype infective juveniles, paratype females, and second-generation paratype males and females were deposited in the nematode collection of the Museum and Institute of Zoology, Polish Academy of Sciences, Wilcza 64, Warsaw, Poland (see Table S2 for deposition numbers).

**Table S2.** *Steinernema sandneri* n. sp. – permanent slides description and designation numbers in the collection of Museum and Institute of Zoology, Polish Academy of Sciences, Warsaw, Poland.

Slide description	Slide ID
Slide no. 1 – *Steinernema sandneri* n. sp. (Rhabditida: Steinernematidae) (male), holotype, natural host unknown, isolated from soil samples: 51°46’55”N 22°42’35”E in 2017	MIZ PAN WARSZAWA 2-2021/1
Slide no. 2 – *Steinernema sandneri* n. sp. (Rhabditida: Steinernematidae), 55 infective juveniles, paratype, natural host unknown, isolated from soil samples: 51°46’55”N 22°42’35”E in 2017	MIZ PAN WARSZAWA 2-2021/2
Slide no. 3 – *Steinernema sandneri* n. sp. (Rhabditida: Steinernematidae), 36 infective juveniles, paratype, natural host unknown, isolated from soil samples: 51°46’55”N 22°42’35”E in 2017	MIZ PAN WARSZAWA 2-2021/3
Slide no. 4 – *Steinernema sandneri* n. sp. (Rhabditida: Steinernematidae), 10 (males), first generation, paratype, natural host unknown, isolated from soil samples: 51°46’55”N 22°42’35”E, in 2017	MIZ PAN WARSZAWA 2-2021/4
Slide no. 5 – *Steinernema sandneri* n. sp. (Rhabditida: Steinernematidae), 10 (males), first generation, paratype, natural host unknown, isolated from soil samples: 51°46’55”N 22°42’35”E, in 2017	MIZ PAN WARSZAWA 2-2021/5
Slide no. 6 – *Steinernema sandneri* n. sp. (Rhabditida: Steinernematidae), 10 (males), first generation, paratype, natural host unknown, isolated from soil samples: 51°46’55”N 22°42’35”E, in 2017	MIZ PAN WARSZAWA 2-2021/6
Slide no. 7 – *Steinernema sandneri* n. sp. (Rhabditida: Steinernematidae), 10 (males), first generation, paratype, natural host unknown, isolated from soil samples: 51°46’55”N 22°42’35”E, in 2017	MIZ PAN WARSZAWA 2-2021/7
Slide no. 8 – *Steinernema sandneri* n. sp. (Rhabditida: Steinernematidae), 10 (males), first generation, paratype, natural host unknown, isolated from soil samples: 51°46’55”N 22°42’35”E in 2017	MIZ PAN WARSZAWA 2-2021/8
Slide no. 9 – *Steinernema sandneri* n. sp. (Rhabditida: Steinernematidae), 5 (females), first generation, paratype, natural host unknown, isolated from soil samples: 51°46’55”N 22°42’35”E in 2017	MIZ PAN WARSZAWA 2-2021/9
Slide no. 10 – *Steinernema sandneri* n. sp. (Rhabditida: Steinernematidae), 5 (females), first generation, paratype, natural host unknown, isolated from soil samples: 51°46’55”N 22°42’35”E in 2017	MIZ PAN WARSZAWA 2-2021/10
Slide no. 11 – *Steinernema sandneri* n. sp. (Rhabditida: Steinernematidae), 5 (females), first generation, paratype, natural host unknown, isolated from soil samples: 51°46’55”N 22°42’35”E in 2017	MIZ PAN WARSZAWA 2-2021/11
Slide no. 12 – *Steinernema sandneri* n. sp. (Rhabditida: Steinernematidae), 5 (females), first generation, paratype, natural host unknown, isolated from soil samples: 51°46’55”N 22°42’35”E, in 2017	MIZ PAN WARSZAWA 2-2021/12
Slide no. 13 – *Steinernema sandneri* n. sp. (Rhabditida: Steinernematidae), 5 (females), first generation, paratype, natural host unknown, isolated from soil samples: 51°46’55”N 22°42’35”E in 2017	MIZ PAN WARSZAWA 2-2021/13
Slide no. 14 – *Steinernema sandneri* n. sp. (Rhabditida: Steinernematidae), 13 (females), second generation, paratype, natural host unknown, isolated from soil samples: 51°46’55”N 22°42’35”E in 2017	MIZ PAN WARSZAWA 2-2021/14
Slide no. 15 – *Steinernema sandneri* n. sp. (Rhabditida: Steinernematidae), 15 (females), second generation, paratype, natural host unknown, isolated from soil samples: 51°46’55”N 22°42’35”E in 2017	MIZ PAN WARSZAWA 2-2021/15
Slide no. 16 – *Steinernema sandneri* n. sp. (Rhabditida: Steinernematidae), 9 (females), second generation, paratype, natural host unknown, isolated from soil samples: 51°46’55”N 22°42’35”E in 2017	MIZ PAN WARSZAWA 2-2021/16
Slide no. 17 – *Steinernema sandneri* n. sp. (Rhabditida: Steinernematidae), 13 (males), second generation, paratype, natural host unknown, isolated from soil samples: 51°46’55”N 22°42’35”E in 2017	MIZ PAN WARSZAWA 2-2021/17
Slide no. 18 – *Steinernema sandneri* n. sp. (Rhabditida: Steinernematidae), 15 (males), second generation, paratype, natural host unknown, isolated from soil samples: 51°46’55”N 22°42’35”E in 2017	MIZ PAN WARSZAWA 2-2021/18
Slide no. 19 – *Steinernema sandneri* n. sp. (Rhabditida: Steinernematidae), 15 (males), second generation, paratype, natural host unknown, isolated from soil samples: 51°46’55”N 22°42’35”E in 2017	MIZ PAN WARSZAWA 2-2021/19

#### Molecular characterization and phylogenetic relationships

*S. sandneri* n. sp. was characterized genetically by the sequences of the ITS rDNA, D2D3 of 28 S rDNA, and the mitochondrial *cox*1 gene. No variation in the sequences of these genes was found between the analyzed individuals. The D2D3, ITS, and *cox*1 sequences of *S. sandneri* n. sp. were deposited in the GenBank with accession numbers MW078535, MW078536, and MW078544, respectively.

As it is known that the molecular diversity in the group of nematodes assigned as *S. kraussei* is relatively high, we included multiple *S. kraussei* sequences in the molecular analysis, also these of *S. kraussei* from the Lublin region, which are sympatric to the new species (Table S1). Compared to other species of the genus *Steinernema* with ITS sequences available in the GenBank, *S. sandneri* S17-050 showed the highest ITS sequence identity with *S. kraussei* strains, i.e. 96.0–97.7%, corresponding to 16–28 nucleotide substitutions (Table S3). It was also noted that the GeneBank sequence AY171250, attributed to *S. kraussei* from Belgium, displayed 99.7% identity and 2 bp difference from this of S17-050 isolate, which implies that this nematode is a conspecific to *S. sandneri* n. sp and should be considered as misidentification. Among the other *Steinernema* species, the most similar sequence of the ITS region with *S. sandneri* n. sp. was displayed by *S. silvaticum* (94.5–95.0% identity, 34–37 substitutions) and *S. xinbinense* (94.5% identity, 35 nucleotide substitutions). The ITS sequences of the other species of the *Steinernema* genus were more divergent from that of *S. sandneri* n. sp., showing identity ≤ 94% and at least 41 nucleotide substitutions (Tables 4 and S3).

**Table S3.** Percentage of similarity (upper triangle) and genetic distance measured by the number of nucleotide substitutions (lower triangle) in the sequences of ITS rDNA regions of *S. sandneri* n. sp., *S. kraussei* and *S. silvaticum* isolates, the closest relatives.

	Species	Acc. no.	1	2	3	4	5	6	7	8	9	10	11	12	13	14	15	16
1	*S. sandneri*	MW078536	–	97.0	97.7	97.7	97.5	97.5	97.2	97.7	97.5	96.6	96.5	96.3	96.0	95.0	94.9	94.5
2	*S. kraussei*	AY230174	21	–	98.1	98.1	97.9	97.9	98.1	97.2	95.7	98.1	98.2	97.5	97.5	95.4	95.3	94.9
3	*S. kraussei*	AY171270	17	13	–	99.2	98.5	98.5	99.6	98.6	98.2	100	99.3	99.4	98.3	96.1	96.0	95.6
4	*S. kraussei*	AY171248	16	12	5	–	99.0	99.0	99,0	99.0	97.3	99.2	98.9	98.6	97.6	95.7	95.6	95.2
5	S. kraussei*	KY819012	18	14	11	6	–	99.7	98.2	99.6	96.6	98.5	98.9	97.9	97.2	95.3	95.2	94.8
6	*S. kraussei*	AY171264	18	14	11	6	2	–	98.2	99.9	86.6	98.5	99.2	97.9	96.9	95.3	95.2	94.8
7	*S. kraussei*	AB243442	20	13	3	6	13	13	–	98.2	97.8	99.6	98.9	99.0	98.3	96.3	96.1	95.7
8	*S. kraussei*	AY230175	17	14	10	6	3	1	13	–	96.8	98.6	99.3	98.1	96.9	95.5	95.3	94.9
9	*S. kraussei*	JN683825	17	14	1	6	12	12	4	11	–	99.2	98.5	99.5	98.3	96.1	96.0	95.6
10	*S. kraussei*	AY171258	17	13	0	5	11	11	3	10	1	–	99.3	98.6	98.3	96.1	96.0	95.6
11	*S. kraussei*	AY230176	18	12	5	7	8	6	8	5	6	5	–	98.0	97.6	95.6	95.5	95.0
12	S. kraussei*	MW647848	20	16	3	8	14	14	6	13	4	3	8	–	97.8	95.6	95.5	95.0
13	*S. kraussei*	AY230161	28	15	11	15	19	21	11	21	10	11	16	14	–	95.3	95.1	94.7
14	*S. silvaticum*	MG543845	34	30	26	28	32	32	25	31	24	26	30	29	31	–	99.9	99.4
15	*S. silvaticum*	AY171255	35	31	27	29	33	33	26	32	25	27	31	30	32	2	–	99.6
16	*S. silvaticum*	AY230162	37	33	29	31	35	35	28	34	26	29	32	32	34	3	2	–

**Table 4.** Percentage of similarity (upper triangle) and genetic distance measured by the number of nucleotide substitutions (lower triangle) in the sequences of ITS rDNA of *S. sandneri* n. sp. and other closely related *Steinernema* spp.

	Species	Acc. no.	1	2	3	4	5	6	7	8	9	10	11	12	13	14	15	16
1	***S. sandneri*** **n. sp.**	**MW078536**	–	**97.0**	**94.5**	**92.4**	**90.2**	**94.1**	**94.1**	**94.5**	**89.6**	**89.8**	**89.0**	**89.4**	**90.3**	**88.7**	**85.8**	**76.6**
2	*S. kraussei*	AY230174	**21**	–	95.2	92.1	90.4	90.5	93.8	94.9	88.6	89.2	88.6	89.1	89.4	88.0	85.2	76.0
3	*S. silvaticum*	AY230162	**37**	31	–	92.0	90.4	90.2	92.6	94.6	88.8	89.4	89.2	88.9	89.6	88.0	84.8	75.8
4	*S. cholashanense*	EF431959	**51**	48	50	–	94.2	97.2	93.2	94.2	91.3	92.7	91.7	92.8	92.2	91.8	86.6	77.5
5	*S. oregonense*	AF122019	**59**	56	59	33	–	93.5	92.0	92.1	89.9	91.5	89.7	91.2	90.4	90.4	86.8	76.6
6	*S. xueshanense*	FJ660052	**67**	59	63	17	41	–	91.3	92.1	90.3	91.2	89.0	91.2	89.8	89.9	85.6	76.9
7	*S. tielingense*	GU994201	**41**	42	51	42	48	56	–	94.8	89.9	89.5	89.7	90.5	90.1	89.2	86.2	77.2
8	*S. xinbinense*	JN171593	**35**	34	36	35	47	50	34	–	90.8	90.8	90.2	91.5	91.0	90.6	86.6	77.0
9	*S. feltiae*	AF121050	**73**	73	74	57	60	61	68	60	–	95.8	91.4	94.0	93.4	94.4	88.9	76.1
10	*S. ichnusae*	EU421129	**71**	70	71	50	52	55	71	62	28	–	91.8	95.5	93.9	85.2	88.6	76.9
11	*S. jollieti*	AY171265	**76**	73	70	53	61	62	67	63	54	46	–	91.1	90.0	91.1	86.1	77.3
12	*S. weiseri*	AY171268	**72**	69	73	48	52	55	62	55	39	32	51	–	94.5	96.6	89.0	77.0
13	*S. nguyeni*	KP325084	**67**	69	70	53	60	61	67	61	47	43	56	39	–	92.9	87.9	76.4
14	*S. litorale*	AB243441	**78**	78	80	57	59	61	73	63	38	34	46	24	44	–	88.7	77.2
15	*S. hebeiense*	DQ105794	**98**	97	102	92	84	90	93	90	76	80	82	77	81	74	–	74.9
16	*S. monticolum*	AF122017	**140**	139	142	133	134	133	132	134	143	135	120	134	132	132	143	–

The highest sequence identity of the D2D3 region of the new species was 98.2%, corresponding to 15 nucleotide substitution, in respect to the analyzed *S. kraussei* strains. The new species differs from other species from the *feltiae-kraussei* group by at least 21 bp, showing ≤ 97.5% nucleotide identity (Table 5).

**Table 5.** Percentage of similarity (upper triangle) and genetic distance measured by the number of nucleotide substitutions (lower triangle) in the sequences of D2D3 domain of 28 S rDNA of *S. sandneri* n. sp. and other closely related *Steinernema* spp.

	Species	Acc. no.	1	2	3	4	5	6	7	8	9	10	11	12	13	14
1	***S. sandneri*** **n. sp.**	**MW078535**	–	**98.2**	**98.2**	**98.2**	**96.2**	**97.5**	**97.5**	**96.8**	**96.8**	**96.6**	**96.1**	**97.0**	**95.7**	**92.4**
2	*S. kraussei*	AF331896	**15**	–	99.8	99.8	97.2	98.2	98.9	97.6	98.2	97.9	97.4	98.1	96.2	92.6
3	*S. kraussei*	GU569053	**15**	2	–	99.5	97.2	98.6	99.1	97.8	98.5	98.1	97.6	98.1	96.6	92.7
4	*S. kraussei* *	MW647849	**15**	2	4	–	97.2	98.2	98.9	97.7	98.2	97.8	97.3	97.0	95.7	92.9
5	*S. silvaticum*	MG547576	**32**	24	24	24	–	97.2	96.8	96.1	96.6	96.1	95.7	96.7	94.7	91.1
6	*S. cholashanense*	EF520284	**21**	14	12	14	24	–	98.4	97.5	98.1	97.7	97.2	97.8	96.4	92.7
7	*S. oregonense*	AF331891	**21**	9	7	11	27	14	–	97.9	98.7	98.2	98.0	98.6	96.9	93.4
8	*S. xueshanense*	FJ666053	**27**	19	19	19	33	20	18	–	98.0	97.9	97.3	98.1	96.6	92.7
9	*S. feltiae*	AF3311906	**27**	15	13	17	29	16	11	17	–	99.3	98.8	99.4	97.2	93.3
10	*S. ichnusae*	EU421130	**28**	18	16	18	32	19	14	17	5	–	98.6	98.9	97.2	93.3
11	*S. jollieti*	GU569051	**32**	22	20	24	36	23	16	22	9	12	–	98.9	96.7	93.1
12	*S. weiseri*	GU569059	**26**	16	16	18	28	19	12	16	5	8	9	–	97.1	93.7
13	*S. texanum*	EF152569	**37**	31	29	31	45	30	26	28	24	23	27	25	–	93.1
14	*S. monticolum*	EF439651	**56**	53	53	54	69	56	49	56	51	52	54	49	53	–

Note: * *S. kraussei* strain sympatric to *S. sandneri* n. sp.

The analysis of the *cox*1 gene sequences showed 92.9–93.8% identity to the sequences of *S. kraussei* isolates (36–40 bp difference) and ≤ 87.7% identity (minimum 70 bp difference) to other sequences of *Steinernema* spp. (Table 6).

**Table 6.** Percentage of similarity (upper triangle) and genetic distance measured by the number of nucleotide substitutions (lower triangle) in the sequences of *cox*1 gene of *S. sandneri* n. sp. and other closely related *Steinernema* spp.

	Species	Acc. no.	1	2	3	4	5	6	7	8	9	10	11
1	***S. sandneri*** **n. sp.**	**MW078544**	–	**93.7**	**93.8**	**92.9**	**87.7**	**86.6**	**88.4**	**84.7**	**86.4**	**85.9**	**85.7**
2	*S. kraussei*	JN683829	**36**	–	94.4	94.0	87.1	86.4	87.3	84.1	85.7	84.5	85.0
3	*S. kraussei*	AY943990	**35**	32	–	95.6	87.8	86.8	86.7	85.0	86.2	85.2	85.4
4	*S. kraussei* *	MW647850	**40**	34	25	–	86.2	86.4	86.9	83.6	85.7	84.5	85.4
5	*S. silvaticum*	MG547572	**70**	73	68	78	–	84.7	85.4	83.6	83.5	82.9	84.7
6	*S. oregonense*	AY943995	**76**	77	75	77	87	–	87.8	85.5	86.6	83.4	86.8
7	*S. feltiae*	JQ423217	**66**	72	75	74	83	69	–	85.9	88.0	84.0	96.9
8	*S. jollieti*	GU569068	**87**	90	85	93	93	82	80	–	85.0	84.7	84.1
9	*S. weiseri*	GU569075	**77**	81	78	81	99	76	68	85	–	82.7	84.8
10	*S. kushidai*	AY943991	**80**	88	84	88	97	94	91	87	98	–	84.5
11	*S. monticolum*	AY943994	**81**	85	83	83	87	75	74	90	86	88	–

Note: * *S. kraussei* strain sympatric to *S. sandneri* n. sp.

**Figure 6:**
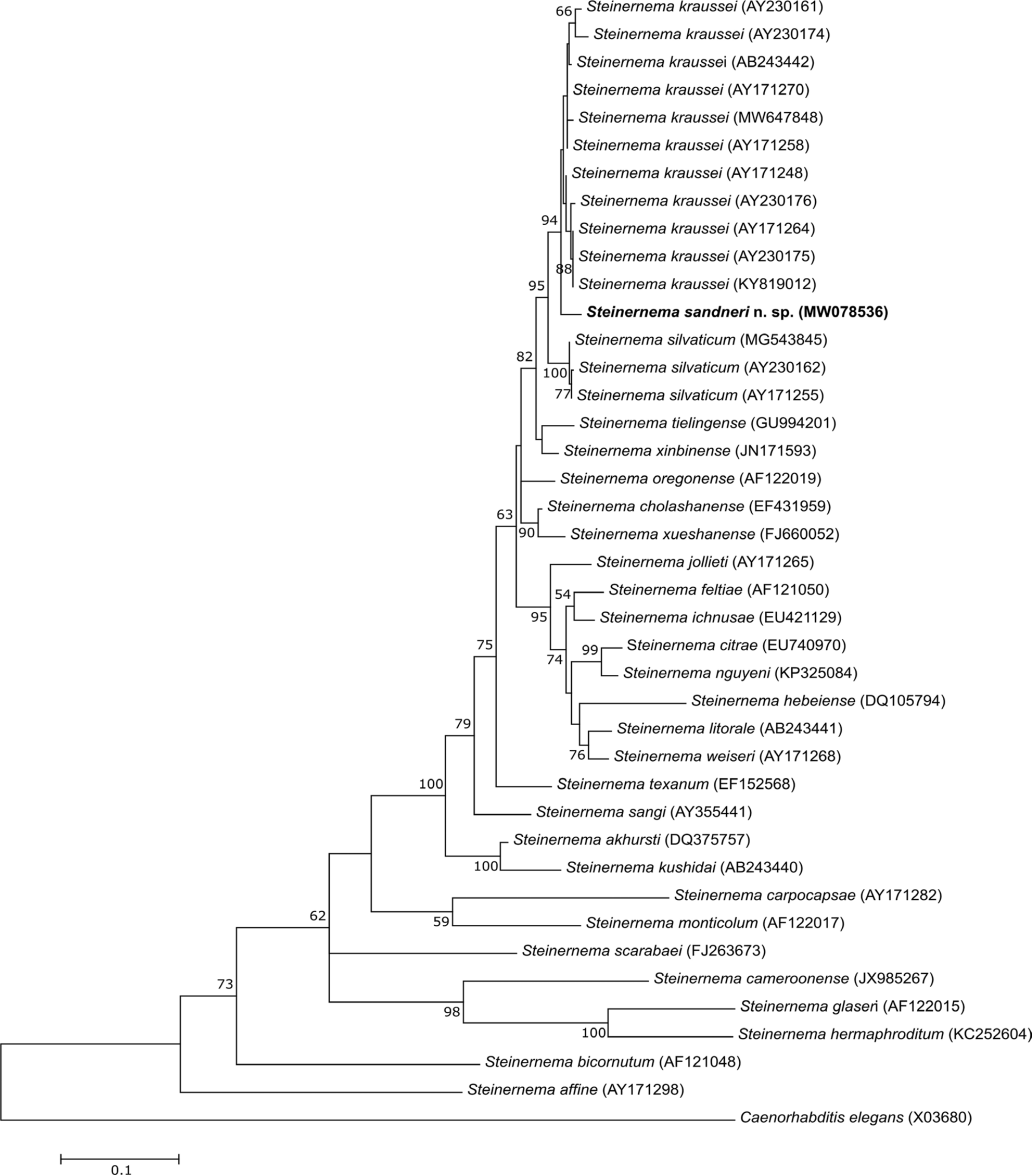
Phylogenetic tree of the phylogenetic relationships of *S. sandneri* n. sp. with other species of the genus *Steinernema* based on sequences of the ITS rDNA. Bootstrap values > 50% are indicated at the branching points. The scale bar indicates the number of nucleotide substitutions per site. The evolutionary history was inferred using the Maximum Likelihood method based on the HKY + G model. All positions containing gaps were eliminated. There were a total of 646 positions in the final dataset. Evolutionary analyses were conducted in MEGA6.

The alignment of the analyzed ITS sequences resulted in 870 positions, in which 266 positions were conserved, while 573 positions were variable, including 426 parsimony-informative and 136 singleton ones. The phylogenetic tree based on the ITS sequences shows that *S. sandneri* n. sp.*, S. kraussei*, and *S. silvaticum* form a monophyletic cluster with 95% bootstrap support (BS) within the *feltiae-kraussei* group. It is also noted that *S. sandneri* n. sp. clusters with *S. kraussei* isolates as a sister group with 94% BS (Fig. 6). The ITS rDNA of *S. sandneri* n. sp. differs from that of the other species of the *feltiae-kraussei* group by four unique traits (present in the sequence alignment only in the new species but not in the others from the group) in the following positions: 530, 608, 656, and 713. In addition, *S. sandneri* n. sp. contains a unique stretch of eight adenine nucleotides in position 480–487 of the ITS sequence.

**Figure 7:**
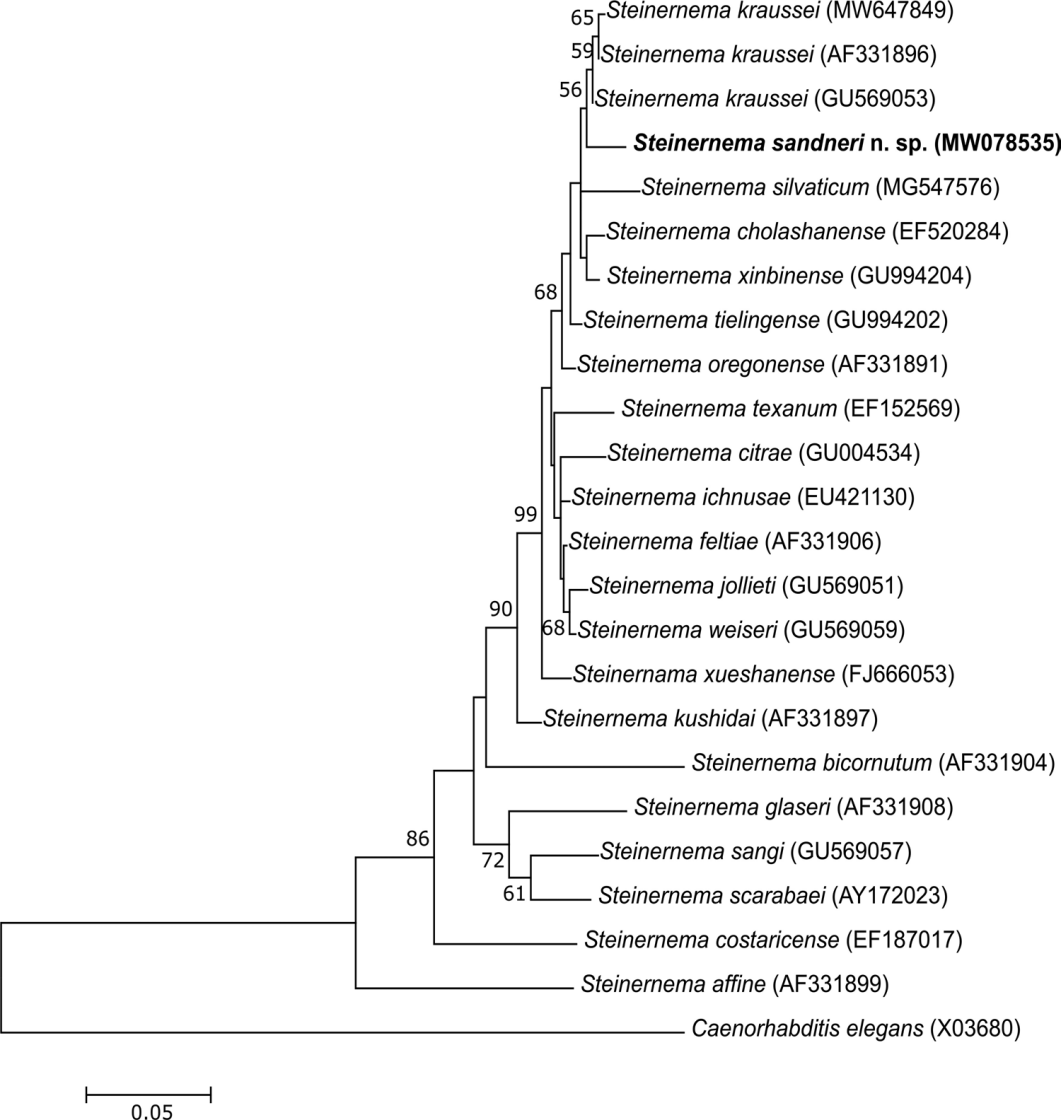
Phylogenetic tree of the phylogenetic relationships of *S. sandneri* n. sp. with other species of the genus *Steinernema* based on sequences of the D2D3 regions. Bootstrap values > 50% are indicated at the branching points. The scale bar indicates the number of nucleotide substitutions per site. The evolutionary history was inferred using the Maximum Likelihood method based on the GTR + G model. All positions containing gaps were eliminated. There were a total of 850 positions in the final dataset. Evolutionary analyses were conducted in MEGA6.

In the case of the D2D3 region sequences, the alignment resulted in 899 positions, in which 611 positions were constant, while 247 positions were variable, including 130 parsimony-informative and 117 singleton ones. The phylogenetic tree based on D2D3 sequences shows that *S. sandneri* n. sp. and *S. kraussei* isolates form a monophyletic group with BS 56%, which is a part of the clade comprising *S. silvaticum*, *S. xinbiense*, *S. cholashanense*, *S. tielingense*, and *S. oregonense* with BS 68% (Fig. 7). *S. sandneri* n. sp. differs from the other species of the *feltiae-kraussei* group in the D2D3 region by six diagnostic traits in the following sequence positions: 52, 155, 374, 444, 456, and 463.

**Figure 8:**
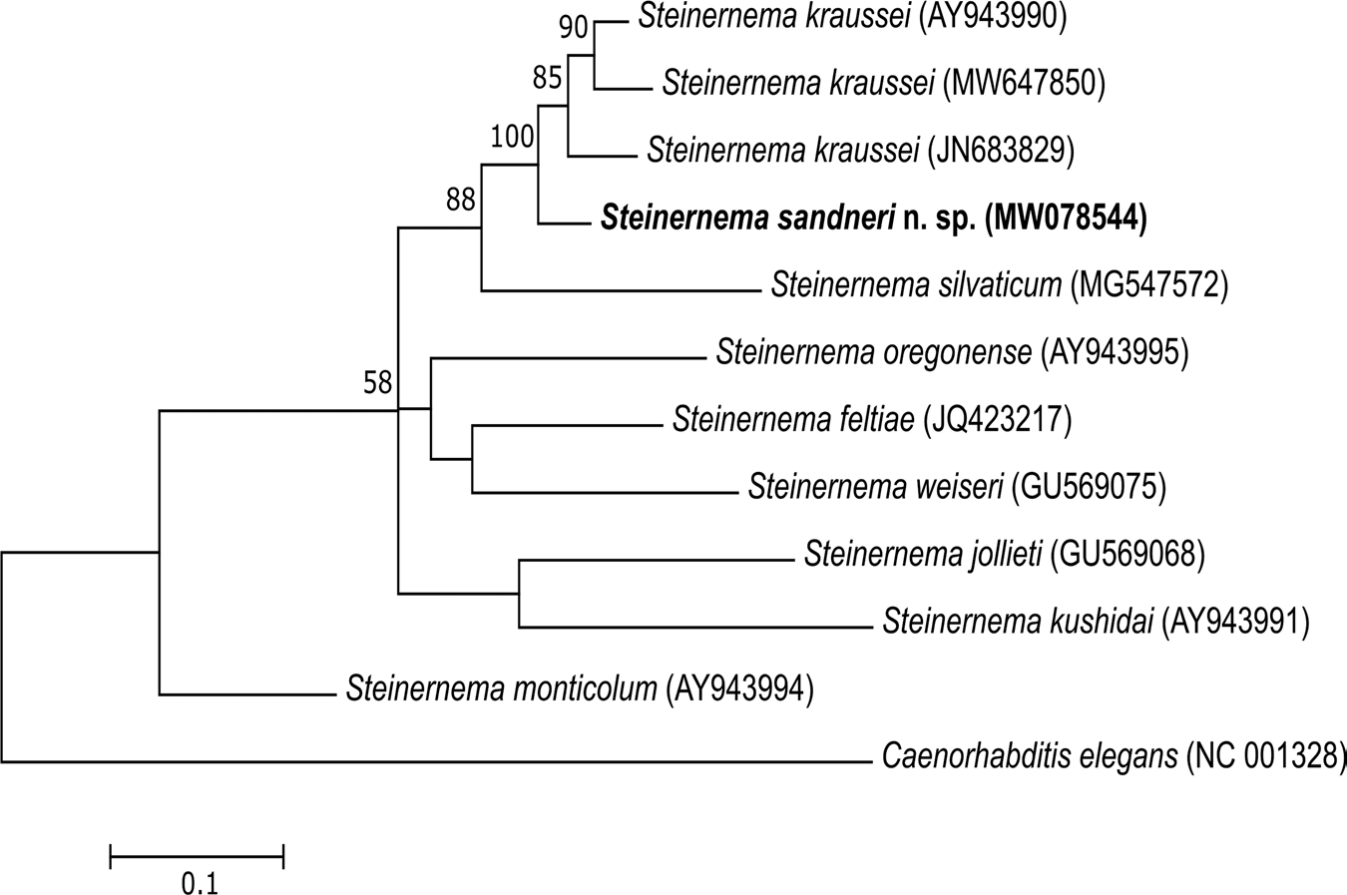
Phylogenetic tree of the phylogenetic relationships of *S. sandneri* n. sp. with other species of the genus *Steinernema* based on *cox*1 gene sequences. Bootstrap values > 50% are indicated at the branching points. The scale bar indicates the number of nucleotide substitutions per site. The evolutionary history was inferred using the Maximum Likelihood method based on the HKY + G + I model. All positions containing gaps were eliminated. There were a total of 567 positions in the final dataset. Evolutionary analyses were conducted in MEGA6.

The alignment in the *cox*1 gene sequences resulted in 567 positions, in which 376 positions were conserved, while 191 positions were variable, including 109 parsimony-informative and 82 singleton ones. The analysis involved only nine steinernematid nematode species, as the number of *cox*1 sequences available in the GeneBank is still limited. The phylogram based on *cox*1 gene sequences shows a clade separating *S. sandneri* n. sp., *S. kraussei*, and *S. silvaticum* with BS 88% (Fig. 8). In this clade, the new species and *S. kraussei* strains form a sister branch with 100% BS. *Steinernema sandneri* n. sp. differs from the other species of the *feltiae-kraussei* group in the *cox*1 gene by five diagnostic traits in positions 21, 138, 165, 225, and 263.

## Discussion

Sequence analysis of ITS rDNA and D2D3 expansion segment of 28 S rDNA have been proved useful for estimation of EPN species, by supporting morphological data (Nadler et al., 2006; Nguyen, 2007a, b; Stock et al., 2001). The phylogenetic trees based on ITS, D2D3, and *cox*1 gene sequences presented in this paper show that *S. sandneri* n. sp. has a unique position in the *feltiae-kraussei* group and is evolutionarily very close to *S. kraussei* and *S. silvaticum.* A number of studies highlighted also the suitability of sequence divergence of these two regions as a good indication of lineage independence (e.g. Spiridonov et al., 2004a, b). The pairwise distances of sequences of the three studied genes clearly differentiate the new species from other nematodes in the *feltiae-kraussei* group. Nevertheless, so far, there is no defined threshold of the ITS or D2D3 rDNA similarity that may indicate whether the studied nematode is a new species or not. Nguyen (2007a, b) suggested an ITS threshold of 95% for *Steinernema* species; however, many closely related species of this genus do not meet this threshold – the difference in ITS sequences between closely related species of *Steinernema* is often ~3% (Spiridonov et al., 2004a, b). The sequence of the ITS region of *S. sandneri* n. sp. shows 2.3–4.0% difference from that of *S. kraussei* isolates (or 3.5–6.0% according to the other sequence identity definition). In fact, the main limitation of using the ITS sequence for estimation of the evolutionary relationships of EPN is their intra-species and intra-individual sequence variability, making sequence aligning dubious and varying the estimation of the sequence identity (Půža et al., 2015).

In turn, phylogenetic analyses of D2D3 have provided evidence that this region has fever ambiguously aligned positions than ITS rDNA, nevertheless it is too conservative to be informative of the relationships between closely related species of the *feltiae-kraussei* group (Nadler et al., 2006). The assessment of the amount of phylogenetic information by determination of the number of variable sites in the sequence alignments used in this study demonstrated that the D2D3 region had a substantially lower number of such positions, compared to ITS rDNA, i.e. 27.5 vs 66.2%. The D2D3 sequence of *S. sandneri* n. sp. shows 1.8% difference from *S. kraussei* and ≥ 2.5% divergence from the other species of the group.

We also analyzed the sequence of the mitochondrial *cox*1 gene of S17-050 nematode. The analysis revealed the highest level of its genetic divergence (6.2–7.1%) from sequences of *S. kraussei* strains, compared to the other molecular markers used. Data have shown that the *cox*1 gene undergoes fast evolution within the *feltiae-kraussei* group, inferring well the phylogenetic relationships among closely related species of this clade (Peat et al., 2009; Szalanski et al., 2000). However, the suitability of this gene to *Steinernema* species delimitation is still limited since a low number of sequences are available for comparison. In the case of the *cox*1 phylogram presented in this study, some uncertainty occurs due to the low number of sequences included; therefore, this is only an approach to resolving evolutionary relationships steinernematid nematode species related to *S. sandneri* n. sp. before more *cox*1 sequences appear.

In addition, the new species is well supported by the molecular diagnostic traits. Current evidence suggests that finding autapomorphies is useful in delimitation of nematode species for better indication of lineage independence (Adams et al., 2007). The sequence alignments of *S. sandneri* n. sp. show that it has four, six, and five diagnostic traits for ITS, D2D3, and *cox*1, respectively. *S. sandneri* n. sp. can also be easily differentiated from the other species from the group by the unique stretch of adenine nucleotides in the sequence of ITS rDNA.

In conclusion, the molecular analysis based on ITS rDNA, D2D3 of 28 S rDNA, and *cox*1 gene sequences confirms the status of *S. sandneri* n. sp. as a new species according to the phylogenetic and evolutionary species concept (Adams, 1998).
